# Supplementary material for: Machine learning-based classification of dual fluorescence signals reveals muscle stem cell fate transitions in response to regenerative niche factors
Source: NPJ Regen Med. 2023 Jan 14;8:4. doi: 10.1038/s41536-023-00277-4 (PMC9839750; doi:10.1038/s41536-023-00277-4)
Supplement: Supplementary file 1 — Supplementary Information [file 41536_2023_277_MOESM1_ESM.pdf]

Machine learning-based classification of dual fluorescence signals reveals muscle stem cell fate transitions in response to regenerative niche factors

Matteo Togninalli*[,1], Andrew T.V. Ho*[,1], Christopher M. Madl*[,1], Colin A. Holbrook[1], Yu Xin Wang[1], Klas E.G. Magnusson[1,2], Anna Kirillova[1], Andrew Chang[1] and Helen M. Blau[1,3]

* These authors contributed equally to this manuscript

[1] Baxter Laboratory for Stem Cell Biology, Department of Microbiology and Immunology, Institute for Stem Cell Biology and Regenerative Medicine, Stanford School of Medicine, Stanford, California 94305-5175, USA

[2] Department of Signal Processing, ACCESS Linnaeus Centre, KTH Royal Institute of Technology, 100 44 Stockholm, Sweden

[3] Corresponding author, hblau@stanford.edu

Supplementary Information

1. Supplementary Figures……………………………………………………………………...2

2. Supplementary Table………………………………………………………………………...7

Supplementary Figures

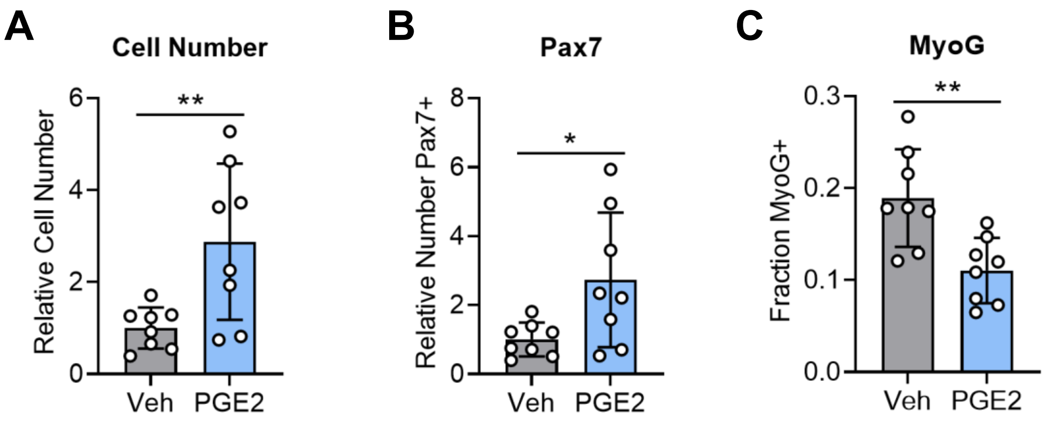

Supplementary Figure 1. Population-level analysis of wild-type PGE2-treated MuSCs. Wild-type MuSCs were treated with PGE2 starting at day 0 and fixed and immunostained at day 7. An increase in **(A)** total cell number and **(B)** the number of Pax7+ cells was observed for PGE2-treated MuSCs compared to vehicle controls. **(C)** PGE2 treatment also reduced the fraction of committed cells staining positive for myogenin compared to vehicle controls. *p<0.05, **p<0.01, two-tailed Student's *t*-test. n = 8 independent replicates. Data are represented as mean ± standard deviation.

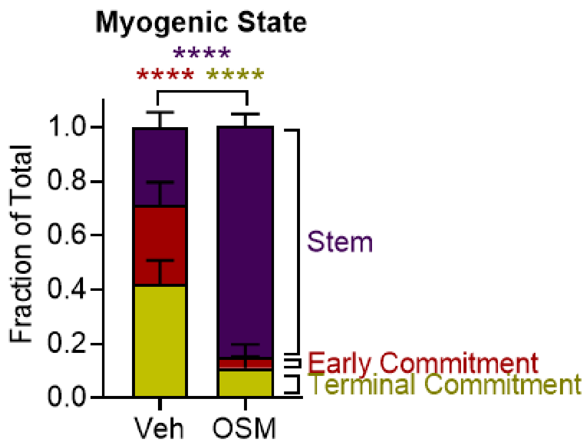

Supplementary Figure 2. Myogenic state for cells treated with OSM starting on day 0 of culture. Similar to treatment starting at day 3, treatment with OSM starting at day 0 resulted in significantly less myogenic commitment and greater maintenance of the stem cell phenotype. ****p<0.0001, two-tailed Student's *t*-test. n = 8 independent replicates for vehicle control and n = 4 for OSM treatment. Data are represented as mean ± standard deviation.

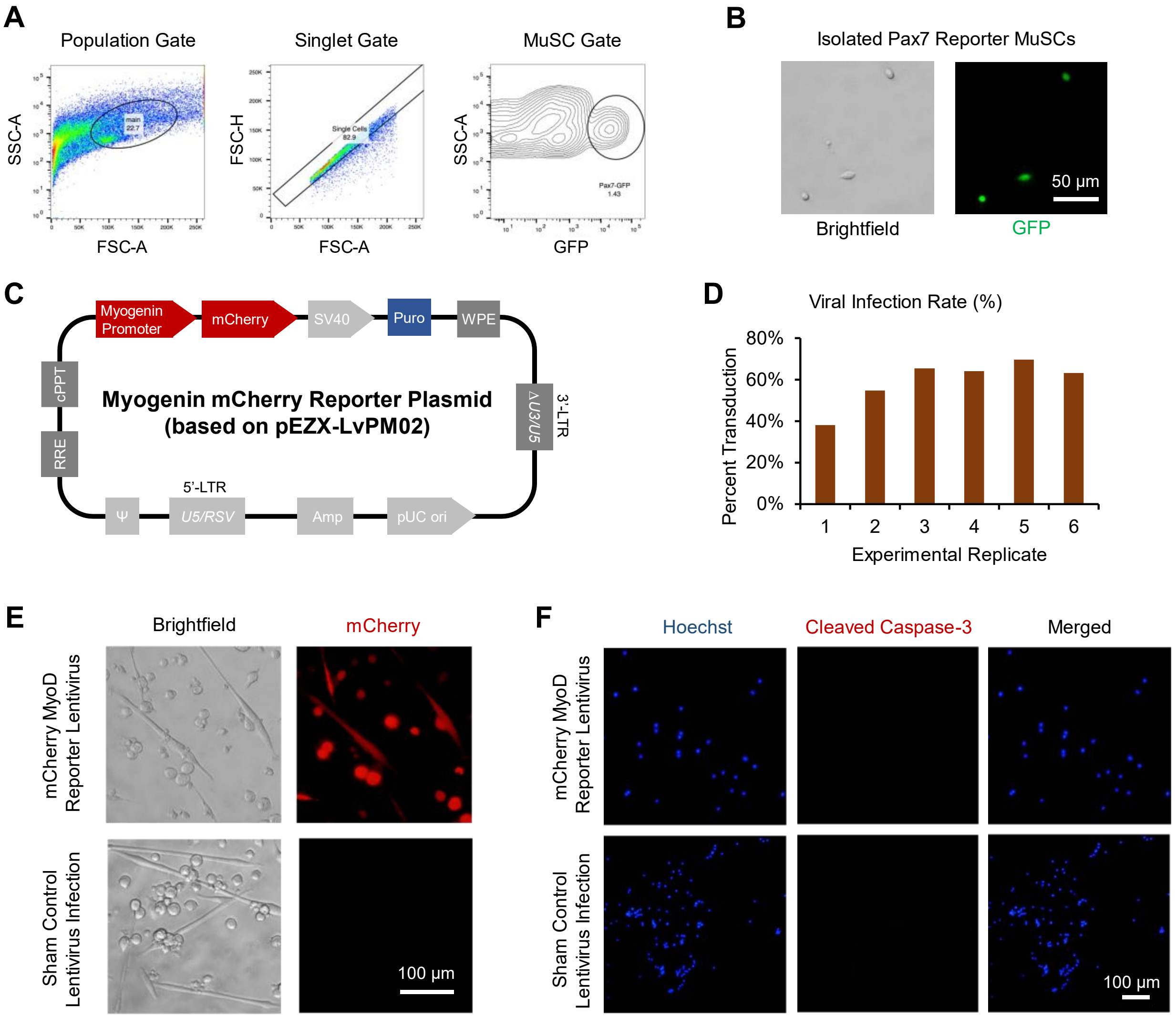

Supplementary Figure 3. Characterization of binary fluorescent reporter MuSCs. (A) Representative FACS gating strategy to enrich for Pax7-eGFP reporter MuSCs. **(B)** Representative brightfield and fluorescence images of sorted Pax7-eGFP reporter MuSCs. **(C)** Plasmid map for the lentiviral vector used to generate the MyoG-mCherry dual reporter MuSCs. **(D)** Viral infection rate across experimental replicates. **(E)** The MyoG-mCherry commitment reporter was validated by inducing differentiation in transduced and sham control MuSCs and measuring mCherry fluorescence by microscopy. **(F)** Transfected MuSCs exhibited negligible viral toxicity as measured by immunofluorescence for the apoptosis marker cleaved caspase-3.

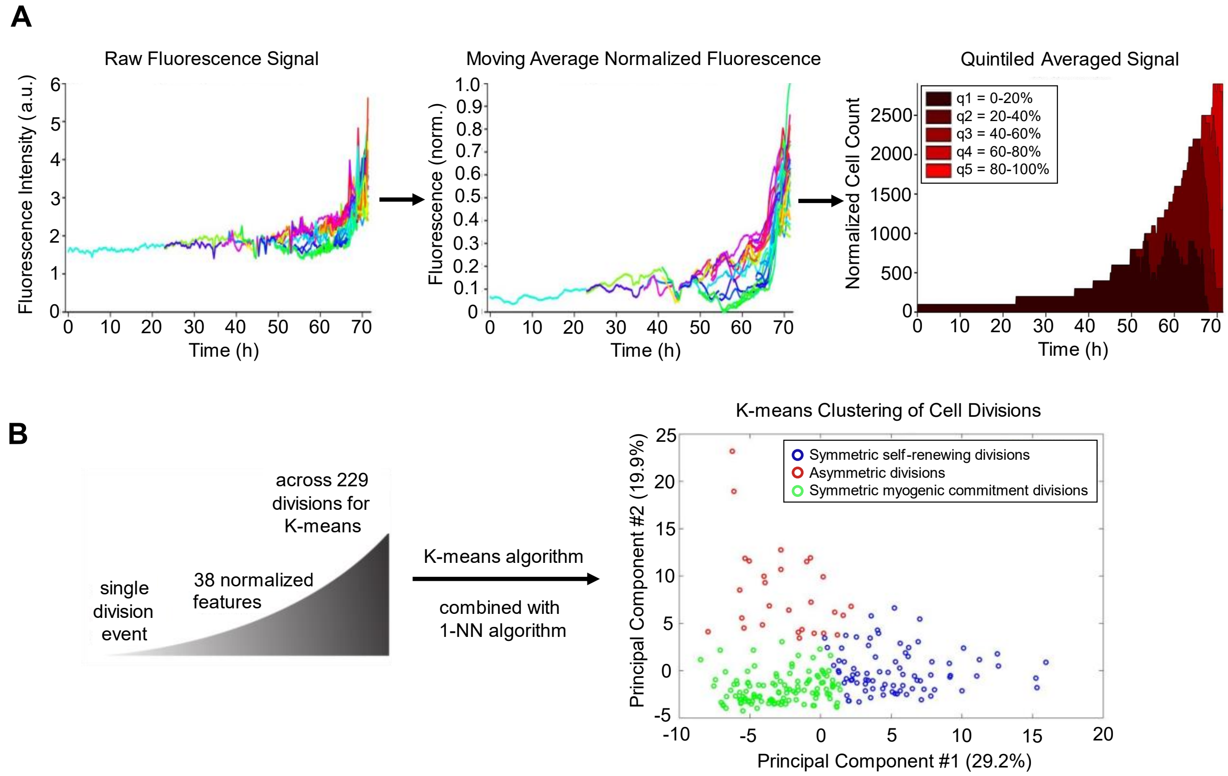

Supplementary Figure 4. Automated analysis of fluorescence signals for division classification. (A) The raw fluorescence intensity signals from a single MuSC and its progeny were normalized to the maximum value in the microwell over the time course experiment and smoothed with a moving average filter. The normalized fluorescence signal was then used to bin the cells into quintiles of reporter expression. **(B)** To classify each division event, 38 normalized features were extracted from the time lapse data (see Supplementary Table S1) and fed into a K-means algorithm to cluster the division features. Cell division assignments were performed using a 1-nearest neighbor (1-NN) classifier.

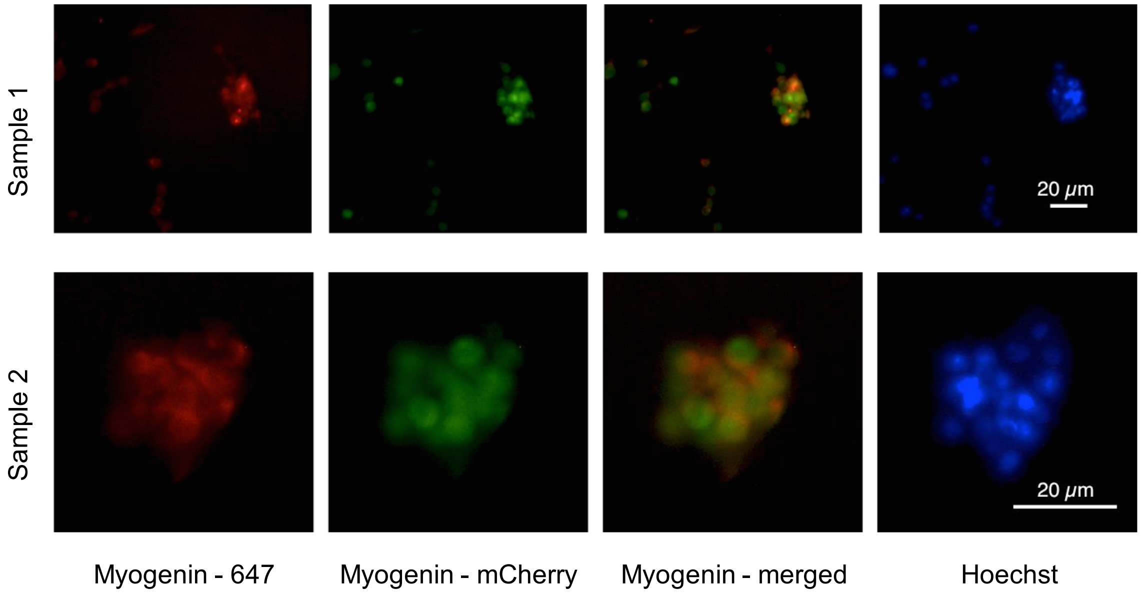

Supplementary Figure 5. Immunofluorescence images of two colonies expressing myogenin post-differentiation at the end point of time-lapse imaging. The red and the green channels correspond to immunostaining for myogenin expression marked with an AlexaFluor 647 secondary antibody and the genetically encoded mCherry reporter, respectively.

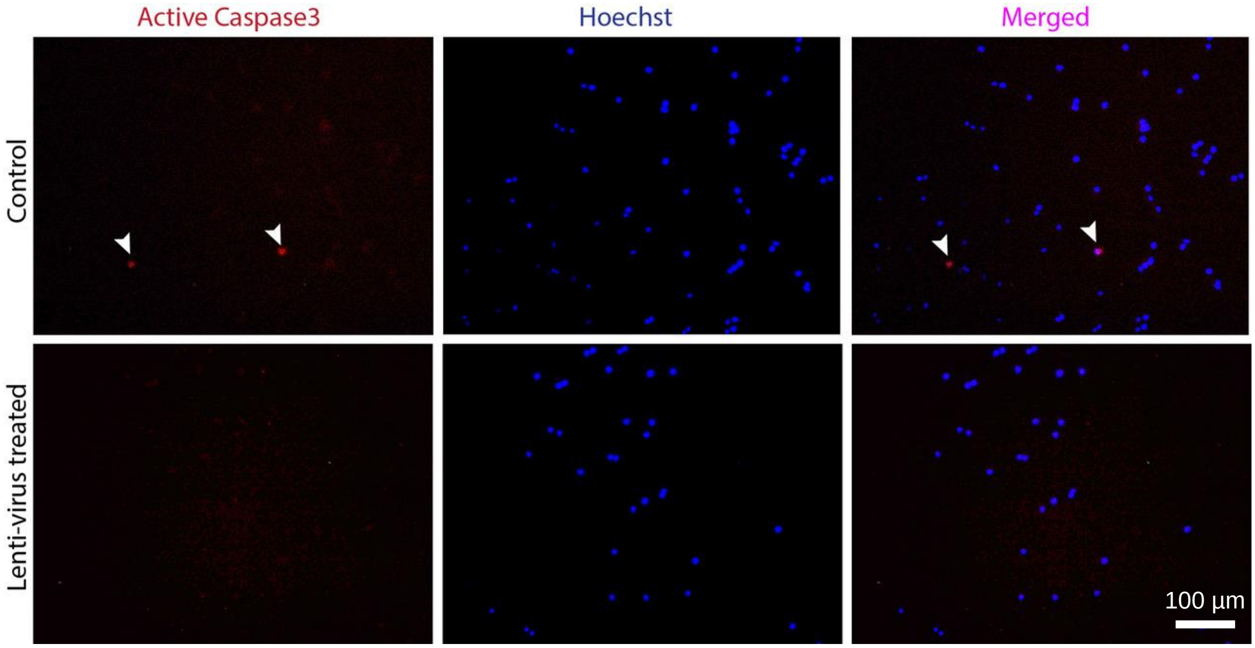

Supplementary Figure 6. Immunofluorescence images of control and virus-treated MuSCs to validate specificity of cleaved caspase-3 antibody staining. The red channel corresponds to staining for cleaved (active) caspase-3, with white arrows marking the active caspase-3 positive cells in the control group.

Supplementary Table

Supplementary Table 1: Extracted features with relative weights for K-means clustering of divisions. The weights were empirically determined by validation with ground truth lineage trees in which the divisions were manually labeled. The fluorescence quantified signals account for 30 features out of a total of 38.

Parameter#	Feature	Weight
**1**	Parent/daughter myogenin min	2
**2**	Parent/daughter myogenin max	2
**3**	Parent/daughter myogenin avg	2
**4**	Parent/daughter myogenin variance	2
**5**	Parent/daughter myogenin beginning-end span	2
**6**	Parent/daughter Pax7 min	1
**7**	Parent/daughter Pax7 max	1
**8**	Parent/daughter Pax7 avg	1
**9**	Parent/daughter Pax7 variance	1
**10**	Parent/daughter Pax7 beginning-end span	1
**11**	Daughter cells lifetime difference	3.5
**12**	Daughter 1 of 2 divided	1
**13**	Daughter 1 of 2 died	1
**14**	Daughter cells fate difference (division)	1
**15**	Daughter cells fate difference (death)	1
**16**	Number of generations	1.5
**17**	Parent no division (**ND**)	1
**18**	Parent died	1
**19**	Parent(**A**)-daughter1(**A**)-daughter2(**SR**)	1
**20**	Parent(**A**)-daughter1(**A**)-daughter2(**SC**)	1
**21**	Parent(**A**)-daughter1(**A**)-daughter2(**ND**)	1
**22**	Parent(**A**)-daughter1(**SR**)-daughter2(**A**)	1
**23**	Parent(**A**)-daughter1(**SR**)-daughter2(**SC**)	1
**24**	Parent(**A**)-daughter1(**SR**)-daughter2(**ND**)	1
**25**	Parent(**A**)-daughter1(**SC**)-daughter2(**A**)	1
**26**	Parent(**A**)-daughter1(**SC**)-daughter2(**SR**)	1
**27**	Parent(**A**)-daughter1(**SC**)-daughter2(**ND**)	1
**28**	Parent(**A**)-daughter1(**ND**)-daughter2(**A**)	1
**29**	Parent(**A**)-daughter1(**ND**)-daughter2(**SR**)	1
**30**	Parent(**A**)-daughter1(**ND**)-daughter2(**SC**)	1
**31**	Parent(**SR**)-daughter1(**A**)-daughter2(**A**)	1
**32**	Parent(**SR**)-daughter1(**SR**)-daughter2(**SR**)	1
**33**	Parent(**SR**)-daughter1(**SC**)-daughter2(**SC**)*****	1
**34**	Parent(**SR**)-daughter1(**ND**)-daughter2(**ND**)*****	1
**35**	Parent(**SC**)-daughter1(**A**)-daughter2(**A**)*****	1
**36**	Parent(**SC**)-daughter1(**SR**)-daughter2(**SR**)*****	1
**37**	Parent(**SC**)-daughter1(**SC**)-daughter2(**SC**)	1
**38**	Parent(**SC**)-daughter1(**ND**)-daughter2(**ND**)	1
